# Secretome analysis of chondroitin sulfate-treated chondrocytes reveals anti-angiogenic, anti-inflammatory and anti-catabolic properties

**DOI:** 10.1186/ar4040

**Published:** 2012-10-02

**Authors:** Valentina Calamia, Lucía Lourido, Patricia Fernández-Puente, Jesús Mateos, Beatriz Rocha, Eulalia Montell, Josep Vergés, Cristina Ruiz-Romero, Francisco J Blanco

**Affiliations:** 1 Osteoarticular and Aging Research Laboratory, Proteomics Unit - ProteoRed/ISCIII, Rheumatology Division, INIBIC - CHU A Coruña, As Xubias 84, A Coruña 15006, Spain; 2 Medical Department, Bioibérica Pharma, Plaza Francesc Macià 7, Barcelona 08029, Spain; 3 CIBER-BBN-ISCIII, CHU A Coruña, As Xubias 84, A Coruña 15006, Spain

## Abstract

**Introduction:**

Chondroitin sulfate (CS) is a symptomatic slow-acting drug for osteoarthritis (OA) widely used in the clinic. The aim of this work is to find proteins whose secretion from cartilage cells under proinflammatory stimuli (IL-1β) is regulated by CS, employing a novel quantitative proteomic approach.

**Methods:**

Human articular chondrocytes released from three normal cartilages were grown in SILAC medium. When complete incorporation of the heavy isotope was achieved, chondrocytes were stimulated with IL-1β 5 ng/ml with or without CS pretreatment (200 µg/ml). Forty-eight hours later, chondrocyte secretomes were analyzed by nano-scale liquid chromatography-mass spectrometry. Real-time PCR, western blot and immunohistochemistry analyses were employed to confirm some of the results.

**Results:**

We could identify 75 different proteins in the secretome of human articular chondrocytes. Eighteen of these were modulated by CS with statistical significance (six increased and 12 decreased). In normal chondrocytes stimulated with IL-1β, CS reduces inflammation directly by decreasing the presence of several complement components (CFAB, C1S, CO3, and C1R) and also indirectly by increasing proteins such as TNFα-induced protein (TSG6). TSG6 overexpression correlates with a decrease in pro-matrix metalloproteinase activation (observed in MMP1 and MMP3 levels). Finally, we observed a strong CS-dependent increase of an angiogenesis inhibitor, thrombospondin-1.

**Conclusion:**

We have generated a quantitative profile of chondrocyte extracellular protein changes driven by CS in the presence of IL-1β. We have also provided novel evidences of its anti-angiogenic, anti-inflammatory, and anti-catabolic properties. Demonstration of the anti-angiogenic action of CS might provide a novel therapeutic approach for OA targeting.

## Introduction

Osteoarthritis (OA) is one of the most prevalent chronic diseases affecting older people. Although its major feature is the progressive destruction of articular cartilage, it is now accepted that OA is a global disease of the joint, also involving the synovial membrane, subchondral bone and periarticular soft tissues [1]. Effective prevention of the structural damage must be a key objective of new therapeutic approaches to treat OA. However, drugs currently available are predominantly directed towards the symptomatic relief of pain and inflammation, doing little to reduce joint destruction [2].

Until now the pharmacological management of OA has been dominated by nonsteroidal anti-inflammatory drugs and analgesics (mainly paracetamol). However, the use of chondroitin sulfate (CS) by OA patients, alone or in combination with glucosamine sulfate (GS), has been rising globally over the last decade. Both molecules are well recognized as symptomatic slow-acting drugs for OA. Moreover, their application has an excellent safety profile, allowing long-term treatment [3-6]. Nevertheless, recent meta-analysis [7] and large-scale clinical trials [8] have demonstrated variable effects on OA symptoms, yielding conflicting results. For this reason, in 2010 we carried out the first pharmacoproteomic analysis of articular chondrocytes treated with exogenous CS and/or GS [9] with the aim of defining more clearly the effects of GS and CS on cartilage biology. In that work, we performed a classical proteomic approach by two-dimensional electrophoresis and mass spectrometry (MS) to describe the cellular proteome of normal human chondrocytes treated with both drugs, alone or in combination, in the presence of IL-1β, a proinflammatory cytokine that plays a pivotal role in the pathogenesis of OA [10]. A large number of target proteins of CS and GS were described, pointing out the wide range effects of these drugs on fundamental aspects of chondrocyte metabolism but also their alternative mechanisms of action in a system model of OA [9].

Once the utility of proteomics for analyzing the putative intracellular targets of CS and GS in cartilage cells was proved [9], we focused on the subset of chondrocyte extracellular proteins that are essential for cartilage extracellular matrix (ECM) synthesis and turnover processes. Furthermore, secreted proteins may end up in the bloodstream, and thereby may have potential use as non-invasive biomarkers [11]. For these reasons, the chondrocyte secretome has emerged as an attractive starting point for the discovery of new OA drug targets, for the monitoring of clinical trials or for the personalization and optimization of long-term therapies. We recently published the first quantitative study of the secretome of primary human articular chondrocytes (HACs) by chondrocyte metabolic labeling, using an *in vitro *model of inflammation by stimulation with IL-1β [12]. In the present work, we aimed to employ this model to generate a quantitative profile of chondrocyte extracellular protein changes driven by CS in the presence of the proinflammatory stimulus, which might provide novel molecular evidence for CS effects.

## Materials and methods

### Cartilage procurement and processing

Macroscopically normal human knee cartilage from three adult donors (70, 73 and 78 years old) with no history of joint disease was provided by the Tissue Bank and the Autopsy Service at CHU A Coruña for the proteomic analysis. The study was approved by the local ethics committee. Cartilage was processed as previously described [13].

### Primary culture of chondrocytes

HACs were isolated as described previously [9,13]. Briefly, cartilage surfaces were rinsed with saline buffer, and scalpels were used to cut parallel vertical sections 5 mm apart from the cartilage surface to the subchondral bone. These cartilage strips were dissected from the bone, and the tissue was incubated with trypsin at 37°C for 10 minutes and then digested with type IV clostridial collagenase. The release of chondrocytes from cartilage was achieved after 16 hours of digestion in an incubator at 37ºC, 5% carbon dioxide.

### Chondrocyte metabolic labeling and differential treatment of SILAC cell populations

The isolated chondrocytes were recovered and plated at low density in SILAC DMEM-Flex (Invitrogen, Paisley, UK) deficient in arginine (R) and lysine (K) supplemented with 10% dialyzed fetal bovine serum (Gibco, Invitrogen), 4.5 g/l glucose (Sigma, St. Louis, MO, USA), 2 mM L-glutamine (Sigma), 100 units/ml penicillin and 100 µg/ml streptomycin. In the case of light media, standard L-lysine and L-arginine were used, while in the heavy media, isotope-labeled L-lysine (^13^C_6_) and isotope-labeled L-arginine (^13^C_6_,^15^N_4_) were used. For the initial cell expansion, 5×10^4 ^chondrocytes from each donor were seeded in two T-25 cell culture flasks (one grown in light medium and one in heavy medium). At confluence cells were recovered from each culture flask by trypsinization and seeded onto two six-multiwell plates (15×10^4 ^for well) for cell treatment. Chondrocytes were used at week 3 in primary culture, when 100% of labeling was reached. Verification of complete labeling was performed as previously described [12]. Briefly, a small aliquot of cells cultured in the heavy media were subjected to protein extraction. The extracts were then digested with trypsin and analyzed by nano-scale liquid chromatography (LC)-MS to determine the degree of incorporation by looking for the presence of light peptides. Verification of cell type was carried out by real-time PCR for the analysis of type II collagen mRNA expression under the conditions of study.

The chondroitin sulfate employed in this work is of bovine origin, with a CS content of 99.9% and a molecular weight of 15.12 kDa. Other characteristics (viscosity, sulfation sites, and so forth) have been previously detailed elsewhere [14]. Chondrocyte stimulation for the experiments was carried out following procedures previously described by our group, in which CS and IL-1β concentrations in the chondrocyte cultures were optimized for the proteomic studies [9,12]. Briefly, cells were washed thoroughly to remove abundant serum proteins and were cultured in serum-free medium with or without chondroitin sulfate (200 μg/ml; Bioibérica, Barcelona, Spain). Two hours later, IL-1β was added to the culture media (5 ng/ml; Sigma). Finally, conditioned media were collected after 48 hours of culture. Cell viability was assessed by Trypan Blue dye exclusion.

### Processing of conditioned media for analysis by LC-MS

Conditioned media obtained from three different donors were analyzed independently. In addition, the off-gel measurements were performed in duplicate to assess the technical reproducibility of the LC-MS set-up.

Conditioned media were collected, centrifuged and filtered using a 0.2 µm filter to ensure removal of any dead cells. Proteins in the individual medium were precipitated with 0.02% sodium deoxycholate for 10 minutes and then with 10% (v/v) trichloroacetic acid overnight at 4ºC. Precipitates were harvested by centrifugation at 13,000 rpm for 15 minutes at 4°C and then washed twice with ice-cold acetone. The protein pellets were dried in air and then resuspended in 6 M urea, 2 M thiourea and 25 mM ammonium bicarbonate. The protein content of the concentrated media was measured using the Bradford reagent from Sigma. Heavy and light samples were then mixed 1:1, and 4 μg of each mixed sample were in-solution reduced, alkylated and digested with trypsin. Digestion was performed overnight with 12.5 ng/l Sequencing Grade Modified Trypsin (Promega, Madison, WI, USA) at 37ºC. The mixtures were acidified with Trifluoroacetic acid (1% final concentration) to stop the enzymatic reaction. The resulted peptides were desalted and filtered through a C18 microcolumn (NuTip; Glygen, Columbia, MD, USA) and finally eluted from the C18 bed using 70% Acetonitrile/0.1% TFA. The organic component was removed by evaporating in a vacuum centrifuge and the peptides were resuspended in 2% Acetonitrile/0.1% TFA. Then 5 µl were injected into a reversed-phase column (Integrafit C18, Proteopep™ II; New Objective, Woburn, MA, USA) for nano-flow LC analysis, using a Tempo nanoLC (Eksigent, Dublin, CA, USA) equipped with a Sun Collect MALDI Spotter/Micro-Fraction Collector (SunChrom GmbH, Friedrichsdorf, Germany).

### Nano-scale LC-MALDI-MS analysis

LC eluate was deposited onto an Opti-TOF LC MALDI target plate (1,534-spot format; ABSciex, Framingham, MA, USA) with a speed of one spot per 15 seconds. Before spotting, the LC microfractions were mixed with MALDI matrix (3 mg/ml α-cyano-4-hydroxycinnamic acid in 70% Acetonitrile and 0.1% TFA containing 10 fmol/μl angiotensin as internal standard). Peptide-containing LC spots were analyzed in a 4800 MALDI-TOF/TOF instrument (ABSciex) with a 200 Hz repetition rate (Nd:YAG laser). MS full-scan spectra were acquired from 800 to 4,000 *m*/*z*. A total of 1,500 laser shots were accumulated for each time-of-flight MS spectrum at an optimized fixed laser setting. Tandem MS mode was operated with 1 kV collision energy with CID gas (air) over a range of 60 to -20 *m/z *of the precursor mass value. The precursor mass window was 300 ppm (full width at half-maximum) in relative mode. A minimum of 800 and a maximum of 1,500 laser shots were accumulated with laser stop conditions set at 10 product ion peaks of signal-to-noise ratio >100 at an optimized, fixed laser setting with metastable suppressor option on. Data-dependent tandem MS settings included acquisition of up to 20 of the most intense ion signals per spot. If two or more consecutive spots in an LC run with precursor *m*/*z *were within 200 ppm tolerance, the spot with the maximum signal-to-noise ratio was subjected to tandem MS analysis.

### Data analysis

Peptide and protein identification and comparative quantification were performed using the Protein Pilot software vs 3.0 (ABSciex) with Paragon Algorithm. MS/MS data was searched against the UniProt/Swiss-Prot database of protein sequences (August 2010; Swiss-Prot, Geneva, Switzerland), using the following parameters: sample type set as SILAC (Lis+6, Arg+10), cysteine alkylation with Iodoacetamide, urea denaturation, one missed cleavage allowed in trypsin digestion and focus in biological modifications. Only proteins with a threshold >95% confidence (>1.3 unused score) were considered for protein identification. Data were normalized for mixing error by bias corrections.

### Real-time PCR assays

Total RNA was isolated from chondrocytes (5×10^5 ^per well) using Trizol Reagent (Invitrogen, Carlsbad, CA, USA), following the manufacturer's instructions. cDNA was synthesized from 1 μg total RNA, using the Transcriptor First Strand cDNA Synthesis Kit (Roche Applied Science, Mannheim, Germany) in accordance with the manufacturer's instructions, and was analyzed by quantitative real-time PCR. The quantitative real-time PCR assay was performed in the LightCycler 480 instrument (Roche Applied Science) using 96-well plates. Primers for thrombospondin-1 (TSP1), TNFα-induced protein (TSG6) and the housekeeping genes, HPRT1 and RPLP0, were designed using the Universal Probe Library tool from the Roche website [15]. Primer sequences were as follows: TSP1 forward, 5'-GCTGCACTGAGTGTCACTGTC-3'; TSP1 reverse, 5'-TCAGGAACTGTGGCATTGG-3'; TSG6 forward, 5'-GCTAGAGGCAGCCAGAAAAA-3'; TSG6 reverse, 5'-ATCCAACTCTGCCCTTAGCC-3'; HPRT1 forward, 5'-TGACCTTGATTTATTTTGCATACC-3'; HPRT1 reverse, 5'-CGAGCAAGACGTTCAGTCCT-3'; RPLP0 forward, 5'-TCTACAACCCTGAAGTGCTTGAT-3', PRPL0 reverse 5'-CAATCTGCAGACAGACACTGG-3'. The results were analyzed using the LightCycler 480 software release 1.5.0 (Roche Applied Science), which automatically recorded the threshold cycle (Ct). An untreated cell sample (basal) was used as the calibrator; the fold-change for this sample was 1.0. Target gene Ct values were normalized against HPRT1 and RPLP0. Data were analyzed using the 2^-ΔΔCt ^method and expressed as the fold-change of the test sample compared with the basal condition [16].

### Western blot analysis

Western blot analyses were performed utilizing standard procedures. Briefly, 20 μg secreted proteins and 50 μg intracellular proteins were loaded and resolved using 10% SDS-PAGE. The separated proteins were then transferred to polyvinylidene fluoride membranes (Immobilon P; Millipore Co., Bedford, MA, USA) by electroblotting and probed with specific antibodies against TSP1 (Santa Cruz Biotechnology, Santa Cruz, CA), TSG6 (Abnova, Taipei, Taiwan), MMP1 and MMP3 (Abcam, Cambridge, UK). Immunoreactive bands were detected and housekeeping control GAPDH (Sigma). Immunoreactive bands were detected by chemiluminescence using corresponding horseradish peroxidase-conjugated secondary antibodies and enhanced chemiluminescence detection reagents (GE Healthcare, Uppsala, Sweden), and then digitized using the LAS 3000 image analyzer (Fujifilm, Tokyo, Japan). For secretome samples, equivalent loadings were verified by Ponceau Red (Sigma) staining after transference (data not shown). Quantitative changes in band intensities were evaluated using ImageQuant 5.2 software (GE Healthcare).

### Immunohistochemical analysis

Cartilage shavings from three healthy donors different from those selected for the proteomics strategy were cut into 6 mm discs using a sterile biopsy punch, and one disc/well was placed into 96-well plates containing 200 µl serum-free DMEM. Plates were incubated for 48 hours with 200 µg/ml CS in presence of IL-1β (5 ng/ml). Frozen samples were then cut at 4 μm with a cryostat (Leica Microsystems, Barcelona, Spain) for immunohistochemical analysis. Sections were incubated with primary antibody to detect the presence of TSP1 (1:50; Santa Cruz Biotechnology, Heidelberg, Germany). The peroxidase/DAB ChemMate™ DAKO EnVision™ detection kit (Dako, Barcelona, Spain) was used to determine antigen-antibody interactions. Negative staining controls were achieved by omitting the primary mAb. Samples were visualized using an optical microscope.

### Statistical analysis

Each experiment was repeated at least three times. The statistical significance of the differences between mean values was determined using a two-tailed *t*-test, considering *P *≤ 0.05 significant. In the proteomic analysis, normalization tools and the statistical package from Protein Pilot software were employed (ABSciex). We considered statistically significant only those changes with *P *≤ 0.05 and a ratio ≥1.2 (or ≤0.83). Where appropriate, results are expressed as the mean ± standard error.

## Results and discussion

Most CS exists as the sugar chains of aggrecan in the cartilage, and its high water-retaining capacity ensures proper cartilage hydration [17]. However, several data in the literature reveal that the mechanism of action of CS is not limited to the fact that it is part of the aggrecan; *in vivo *studies in animal models and *in vitro *studies with human and animal articular cells suggest that the effects of CS result from a combination of numerous factors [18]. We have performed a gel-free quantitative proteomics experiment for the secretome analysis (cell-conditioned media) of HACs treated with bovine CS (95% purity) in the presence of IL-1β. Although HAC supernatants lack the complexity of the intact cartilage ECM, chondrocyte secretome may represent an attractive subproteome for understanding the chondroprotective action of CS [19,20].

### Secretome profiling of IL-1β and CS-treated HACs

Given the key role of chondrocytes in ECM synthesis and turnover, and also the importance of these mechanisms for tissue maintenance (which are disturbed in OA and other joint diseases), we examined the effect of CS in the subset of proteins secreted by chondrocytes (secretome) in an inflammatory environment (IL-1β). Inflammatory molecules, such as proinflammatory cytokines, are critical mediators of the disturbed metabolism and enhance the catabolism of joint tissue involved in OA pathophysiology [21]. For this purpose, supernatants from IL-1β-stimulated chondrocytes, with or without CS treatment, were collected after 48 hours of incubation and were analyzed. Owing to the low complexity of the secretome samples, we carried out a monodimensional approach: we combined equal amounts of proteins from the experimental conditions to be compared (treated or untreated with CS, both in the presence of the cytokine), and then these samples were digested in solution with trypsin. The correspondent tryptic peptides were separated by LC and the peptides were subsequently eluted and subjected to mass spectrometry analysis (MALDI-MS/MS).

**Figure 1:**
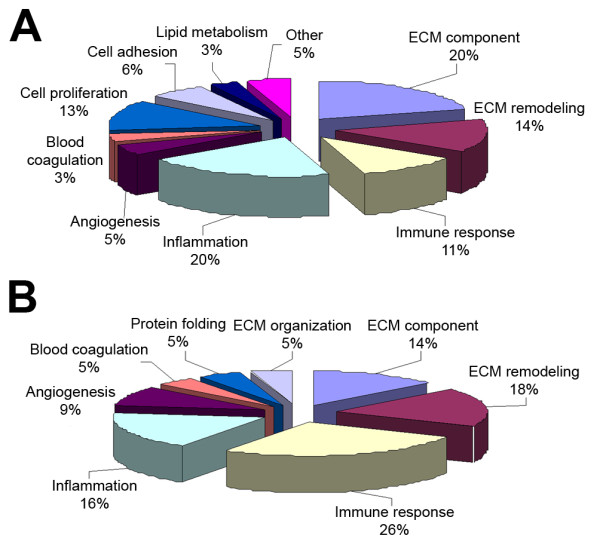
**Bioinformatic analysis of identified and differentially abundant proteins**. Bioinformatic analysis of **(A) **identified and **(B) **differentially abundant proteins according to the Gene Ontology database. The functional distribution graph reveals anti-inflammatory and immunomodulatory properties of chondroitin sulfate, apart from its role in extracellular matrix (ECM) structure.

This procedure resulted in the identification of 75 proteins present in the culture media of IL-1β-treated cells with statistical confidence (73 with Protein Pilot score ≥2). Some of them had not been previously reported to be secreted by chondrocytes [12], but they were found in serum [22] and/or synovial fluid [23] of OA patients and thus possess putative biomarker value. A complete list of these proteins is shown in Table 1. The majority of the identified secreted proteins were cartilage ECM proteins, or proteins with well-established matrix functions. Furthermore, several mediators of the inflammatory response were detected. The molecular function of the identified proteins was categorized by GeneOntology and is shown in Figure 1. The most abundant proteins identified in the samples (in terms of Protein Pilot hits, see Table 1) included well-known cartilage-related proteins, namely fibronectin (FN1) and chitinase-3-like protein 1 (CHI3L1), as well as ECM degradative enzymes, such as stromelysin-1 (MMP3) and interstitial collagenase (MMP1).

**Table 1:** Proteins identified in the secretome of IL-1β-stimulated chondrocytes with or without CS treatment

Symbol	Score	Peptides (95%)	Covariance (%)	Accession number^a^	Name	Function	**OA serum **[22]	**OA synovial fluid **[23]
MMP2	20.24	17	36.4	[P08253]	72 kDa type IV collagenase	Angiogenesis/collagen degradation		x
A1AT	2	2	6	[P01009]	Alpha-1-antitrypsin	Inhibitor of serine proteases		
ANXA2	2	1	36	[P07355]	Annexin A2	Heat-stress response		
B2MG	4	3	26.9	[P61769]	Beta-2-microglobulin	Immunity	x	
PGS1	4.03	3	13.9	[P21810]	Biglycan	Collagen fiber assembly/ECM component		
CATB	3.06	2	15.3	[P07858]	Cathepsin B	Thiol protease		
CCL2	6	4	38.4	[P13500]	C-C motif chemokine 2^b^	Inflammatory response		
CCL8	4	2	38.4	[P80075]	C-C motif chemokine 8^b^	Inflammatory response		
CH3L1	63.26	96	82	[P36222]	Chitinase-3-like protein 1	ECM component		x
CH3L2	2	2	17.7	[Q15782]	Chitinase-3-like protein 2^b^	ECM component		
CLUS	10.09	9	43.2	[P10909]	Clusterin	Immunity	x	x
CCD80	2	1	23.3	[Q76M96]	Coiled-coil domain-containing protein 80	Cell adhesion and matrix assembly		
CO3A1	2.02	1	14.3	[P02461]	Collagen alpha-1(III) chain	ECM component		
CO6A1	4.16	4	28.5	[P12109]	Collagen alpha-1(VI) chain	ECM component		
COCA1	17.35	10	21.1	[Q99715]	Collagen alpha-1(XII) chain	ECM component		
CO1A2	27.65	25	55.2	[P08123]	Collagen alpha-2(I) chain	ECM component		
C1R	8.13	7	31.5	[P00736]	Complement C1r subcomponent	Immunity	x	x
C1S	10.6	9	27.3	[P09871]	Complement C1s subcomponent	Immunity	x	x
CO3	13.4	7	26.4	[P01024]	Complement C3	Immunity	x	
CFAB	8.29	6	17.2	[P00751]	Complement factor B	Immunity	x	x
CXCL3	4.01	4	69.2	[P19876]	C-X-C motif chemokine 3^b^	inflammatory response		
CXCL5	2	1	20.2	[P42830]	C-X-C motif chemokine 5^b^	Inflammatory response		
CXCL6	4.13	5	38.6	[P80162]	C-X-C motif chemokine 6^b^	Inflammatory response		
CYTC	4	3	67.1	[P01034]	Cystatin-C	Inhibitor of cysteine proteinases	x	
PGS2	23.72	22	56	[P07585]	Decorin	ECM component		
DESP	2	1	24.8	[P15924]	Desmoplakin^b^	Cell junction	x	
FBLN3	15.7	12	31.2	[Q12805]	EGF-containing fibulin-like extracellular matrix protein 1	Negative regulator of chondrocyte differentiation	x	x
FINC	152.34	150	58.9	[P02751]	Fibronectin	ECM component	x	x
FBLN1	2	1	11.2	[P23142]	Fibulin-1	Cell adhesion/ECM organization	x	
FSTL1	2.1	2	16.2	[Q12841]	Follistatin-related protein 1	Cell proliferation and differentiation		
GDN	24.73	23	54.5	[P07093]	Glia-derived nexin	Serine protease inhibitor		
GROA	4	5	54.2	[P09341]	Growth-regulated alpha protein	Inflammatory response		
IBP3	12.02	12	57.7	[P17936]	Insulin-like growth factor-binding protein 3	Cell proliferation and differentiation	x	
IBP4	3.36	3	37.6	[P22692]	Insulin-like growth factor-binding protein 4	Cell proliferation and differentiation		
IBP5	4.03	2	55.5	[P24593]	Insulin-like growth factor-binding protein 5	Cell proliferation and differentiation		
IBP6	3.85	3	43.8	[P24592]	Insulin-like growth factor-binding protein 6	Cell proliferation and differentiation	x	
IBP7	2.15	3	29.1	[Q16270]	Insulin-like growth factor-binding protein 7	Cell proliferation and differentiation		
IL6	16	16	42	[P05231]	IL-6	Inflammatory response		
IL8	4.24	6	57.6	[P10145]	IL-8	Inflammatory response		
MMP1	35.12	38	50.1	[P03956]	Interstitial collagenase	Collagen degradation		
K1C10	60.82	36	66.8	[P13645]	Keratin, type I cytoskeletal 10	Intermediate filament	x	x
K1C14	11.36	7	44.9	[P02533]	Keratin, type I cytoskeletal 14^b^	Intermediate filament		
K1C16	15.45	8	48	[P08779]	Keratin, type I cytoskeletal 16^b^	Intermediate filament		
K1C9	30.98	19	60.2	[P35527]	Keratin, type I cytoskeletal 9	Intermediate filament	x	x
K2C1	70.89	41	69.6	[P04264]	Keratin, type II cytoskeletal 1	Intermediate filament	x	x
K22E	43.26	22	69.2	[P35908]	Keratin, type II cytoskeletal 2 epidermal	Intermediate filament	x	x
K2C6B	20.77	11	53.9	[P04259]	Keratin, type II cytoskeletal 6B^b^	Intermediate filament		x
MFGM	6.01	4	20.9	[Q08431]	Lactadherin	Angiogenesis		
LUM	28.15	30	60.7	[P51884]	Lumican	ECM component	x	x
CSF1	4.01	2	17.3	[P09603]	Macrophage colony-stimulating factor 1^b^	Inflammatory response		
TIMP1	17.8	16	57.5	[P01033]	Metalloproteinase inhibitor 1	Metalloprotease inhibitor		x
TIMP2	4	3	32.7	[P16035]	Metalloproteinase inhibitor 2	Metalloprotease inhibitor		
PTX3	2.02	2	14.4	[P26022]	Pentraxin-related protein PTX3	Inflammatory response		
PLTP	5.8	4	31	[P55058]	Phospholipid transfer protein	Lipid transport	x	x
IC1	4	2	18.8	[P05155]	Plasma protease C1 inhibitor	Immunity	x	x
POTEF	2.03	1	27.6	[A5A3E0]	POTE ankyrin domain family member F^b^	Unknown		
SAP	2	1	5	[P07602]	Proactivator polypeptide^b^	Lipid metabolism		
PCOC1	5.93	4	19.8	[Q15113]	Procollagen C-endopeptidase enhancer 1	Collagen metabolism		x
S10A8	1.54	1	35.5	[P05109]	Protein S100-A8^b^	Inflammatory response	x	
S10A9	4	2	34.2	[P06702]	Protein S100-A9^b^	Inflammatory response	x	
PRG4	3.7	3	21.9	[Q92954]	Proteoglycan 4^b^	ECM component	x	x
TRY6	4	2	15.4	[Q8NHM4]	Putative trypsin-6^b^	Serine protease		
NPT2C	2	1	10.9	[Q8N130]	Sodium-dependent phosphate transport protein 2C^b^	Ion transport		
SPRC	2	2	7.3	[P09486]	SPARC	Cell proliferation and differentiation	x	
MMP3	48.64	47	71.9	[P08254]	Stromelysin-1	Collagen degradation		x
QSOX1	4.87	4	16.3	[O00391]	Sulfhydryl oxidase 1	Cell redox homeostasis	x	
SDC4	2	1	13.6	[P31431]	Syndecan-4	ECM component		
TARSH	8.86	9	27.4	[Q7Z7G0]	Target of Nesh-SH3	Cell proliferation and differentiation	x	
TENA	13.36	8	22.5	[P24821]	Tenascin	Cell adhesion/ECM organization		
TETN	4	4	34.2	[P05452]	Tetranectin	Bone mineralization	x	x
TSP1	2.36	2	18.5	[P07996]	Thrombospondin-1	Angiogenesis	x	
BGH3	2	1	22.6	[Q15582]	TGF-beta-induced protein ig-h3	TGF-beta signaling	x	
TSG6	28.35	30	69.3	[P98066]	TNF-inducible gene 6 protein	Cell adhesion		
VCAM1	2	1	12.2	[P19320]	Vascular cell adhesion protein 1	Cell adhesion	x	
VASN	2	2	14.7	[Q6EMK4]	Vasorin	TGF-beta signaling	x	

Proteins identified by SILAC and liquid chromatography-mass spectrometry analysis in the secretome of IL-1β-stimulated chondrocytes with or without chondroitin sulfate treatment. ECM, extracellular matrix; EGF, epidermal growth factor; TGF, transforming growth factor. ^a^Protein accession number according to the SwissProt and TrEMBL databases. ^b^Protein not identified in our previous analysis of chondrocyte secretome [12].

### CS-mediated changes in the chondrocyte secretome

By these means we were able to relatively quantify all the identified proteins with statistical significance. To confirm our findings and exclude the possibility of any quantification differences arising from SILAC labeling [24], the whole experiment was replicated with treatment conditions crossed over (swapping the labeled state of the perturbed cells). Finally, among the identified proteins, 18 presented a significant alteration of their levels due to the pharmacological treatment (six increased and 12 decreased), which are listed in Table 2. We detected the modulation of proteins involved in several processes, such as cartilage ECM structural organization (three proteins, including noncollagenous proteins and proteoglycans), ECM remodeling (four proteins, including proteases and their inhibitors), immune response (six proteins) and angiogenesis (two proteins).

**Table 2:** Extracellular proteins modulated by chondroitin sulfate treatment in IL-1β-stimulated chondrocytes

Accession number^a^	Name	Symbol	Ratio^b^	*P *value	Error factor^c^
[P00751]	Complement factor B	CFAB	0.5566	0.0064	1.397
[Q08431]	Lactadherin	MFGM	0.597	0.0151	1.3855
[Q7Z7G0]	Target of Nesh-SH3	TARSH	0.7017	0.006	1.2209
[P09871]	Complement C1s subcomponent	C1S	0.7085	0.0007	1.1398
[P01024]	Complement C3	CO3	0.7241	0.0023	1.1999
[P02751]	Fibronectin	FINC	0.7321	0.0006	1.1891
[P03956]	Interstitial collagenase	MMP1	0.7113	0	1.0977
[P00736]	Complement C1r subcomponent	C1R	0.7734	0.0071	1.1755
[P08253]	72 kDa type IV collagenase	MMP2	0.7759	0.0001	1.0999
[P36222]	Chitinase-3-like protein 1	CH3L1	0.8088	0.0216	1.4096
[P10909]	Clusterin	CLUS	0.8224	0.05	1.2162
[P08254]	Stromelysin-1	MMP3	0.8256	0.0086	1.1378
[P07093]	Glia-derived nexin	GDN	1.2894	0.0031	1.1664
[O00391]	Sulfhydryl oxidase 1	QSOX1	1.3064	0.0382	1.2709
[Q92954]	Proteoglycan 4	PRG4	1.4393	0.05	1.445
[P98066]	TNF-inducible gene 6 protein	TSG6	1.7773	0.0007	1.3371
[P61769]	Beta-2-microglobulin	B2MG	2.1652	0.0125	1.4553
[P07996]	Thrombospondin-1	TSP1	89.1678	0	>2

Extracellular proteins identified by SILAC and liquid chromatography-mass spectrometry analysis as modulated by CS treatment in IL-1β-stimulated chondrocytes. ^a^Protein accession number according to the SwissProt and TrEMBL databases. ^b^Average SILAC ratios (*n *= 3) that represent the relative protein abundance in CS-treated versus untreated cells, calculated by Protein Pilot 3.0 software (ABSciex). *P *≤ 0.05 was accepted. ^c^Error factor of the quantification (measure of the error in the average quantification ratio as calculated by Protein Pilot 3.0 software).

**Figure 2:**
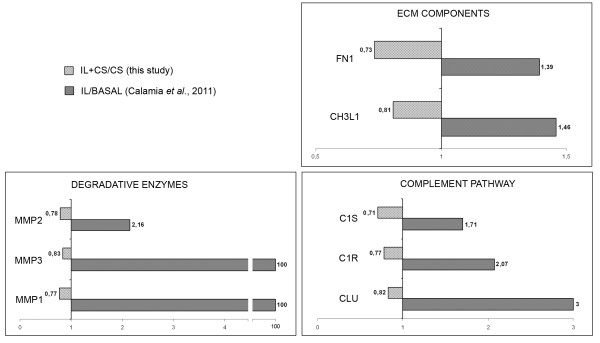
**Role of chondroitin sulfate in counteracting IL-1β-mediated increase of proteins**. Role of chondroitin sulfate (CS) in counteracting IL-1β-mediated increase of proteins related to extracellular matrix (ECM) remodeling, catabolism and inflammation. Graphics show the quantitative modulation caused by CS on this group of proteins (ratio IL+CS/IL, light-gray bars) detected in the present study, and compare these changes with those attributable to IL-1β treatment (ratio IL/BASAL, dark-gray bars) that were found previously [12]. Protein abbreviations are defined in Table 2.

Interestingly, we found distinctively in CS-treated cells a global decrease of immunity-related proteins, degradative enzymes (such as MMP1, MMP2, and MMP3), and some ECM structural proteins (such as fibronectin (FN1) and chitinase-3-like protein 1 (CHI3L1)). Among those proteins described in our previous work as increased by IL-1β [12], which were now decreased by CS, we found FN1 and CHI3L1, two components of normal cartilage matrix (Figure 2). Synthesis and release of both proteins and fragments is often increased in cartilage that is undergoing repair or remodeling, and they have been investigated as markers of cartilage damage in OA [25-27]. Interestingly, the release of FN1 and CHI3L2 from chondrocytes was also detected in a previous proteomic analysis from our group, which aimed to evaluate the differential effect of three distinct CS molecules in chondrocytes [28]. In that work, the presence of these proteins in the chondrocyte secretomes was caused by treatment with a CS of porcine origin, which appeared to trigger catabolic effects in chondrocytes by increasing also the abundance of matrix metalloproteinases (MMP1 and MMP3). On the contrary, treatment with bovine CS (the one employed in the present study) did not have any effect on the release of these four proteins.

### Putative mediators of CS anti-inflammatory and anti-catabolic effects

**Figure 3:**
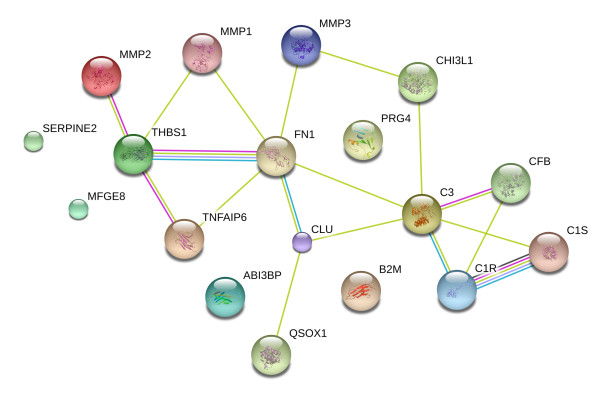
**Protein-protein interaction network of chondroitin sulfate effects**. The STRING database was searched for protein interaction analyses in order to elucidate the effect of chondroitin sulfate on cartilage extracellular matrix (ECM) proteins. As shown, most of the altered proteins interact with each other to constitute a large network. These proteins are involved in several processes, but essentially belong to immune response, inflammation and ECM remodeling pathways.

**Figure 4:**
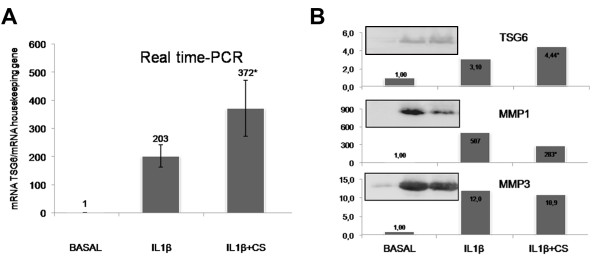
**Chondroitin sulfate-dependent increase of TNFα-induced protein and correlation with the decrease in matrix metalloproteinase activation**. The chondroitin sulfate (CS)-dependent increase of TNFα-induced protein (TSG6 or TNFAIP6), and its correlation with the decrease in matrix metalloproteinase activation. Overexpression values of TSG6 were determined by **(A) **real-time PCR and **(B) **western blot analysis of IL-1β-stimulated human articular chondrocyte secretomes as described in Materials and methods. The CS-mediated decrease of MMP1 and MMP3 activation was confirmed by western blot analysis (B). Each condition was tested in duplicate. Results are expressed as the mean ± standard error of the mean of three independent experiments. Representative images of the western blot assays are shown. *P < 0.05, CS-treated group was significantly different from IL-1β-stimulated group.

We also performed a database search, using STRING software, to visualize protein interactions on the set of CS-modulated proteins and further elucidate its effect on chondrocytes (Figure 3). The role of CS in counteracting the IL-1β-mediated increase of some proteins was also detected for three degradative enzymes and three members of the complement pathway (Figure 2 and Table 2). Recently, a central role for the inflammatory complement system in the pathogenesis of OA has been identified [29]. Expression and activation of complement is abnormally high in human osteoarthritic joints. We show in this study how CS could reduce inflammation directly by decreasing the presence of several complement components (CFAB, CLUS, CO3, C1S and C1R), and also indirectly by increasing proteins such as TSG6. This protein plays a crucial role in ECM formation, inflammatory cell migration and cell proliferation. TSG6 is also a key component of a negative feedback loop operating through the protease network that reduces matrix degradation during the OA process [30]. The mechanism driven by TSG6 leads to a decrease in pro-matrix metalloproteinase activation, which might protect cartilage from extensive degradation even in the presence of acute inflammation (represented in our case by a high level of IL-1β). Western blot analyses were performed to confirm the detected increase of TSG6 caused by CS treatment. As shown in Figure 4, CS increased the amount of TSG6 secreted by chondrocytes, and this increase correlates with a decline in MMP1 and MMP3 levels. These results point to the increase of TSG6 as a putative mediator of the reduction in pro-matrix metalloproteinase activation, suggesting an important role of this mechanism for the anti-catabolic effect of CS.

### Modulation of thrombospondin-1 by CS

A remarkable increase of TSP1, an angiogenesis inhibitor, was detected as a consequence of the CS treatment and counteracting the effect of IL-1β. This result is consistent with our previously observed increase of TSP1 protein driven by CS in the absence of IL-1β stimulation, although in osteoarthritic chondrocytes [28]. TSP1 overexpression reduces inflammation and neovascularization in the OA joint [31]. In our previous study on IL-1β-stimulated chondrocytes, TSP1 presented a ratio of zero [12], indicating a cytokine-dependent dramatic decrease of its release from these cells. IL-1β is a well-recognized angiogenic factor, so the possibility that an increased concentration of IL-1β in OA synovial fluid may reduce the TSP1 expression in severe stages of OA cannot be excluded. The selective inhibition of angiogenesis - also confirmed by the decrease of lactadherin, a protein that promotes vascular endothelial growth factor-dependent neovascularization [32] - demonstrates a novel mechanism of action of CS according to recent results obtained in synoviocytes [33].

**Figure 5:**
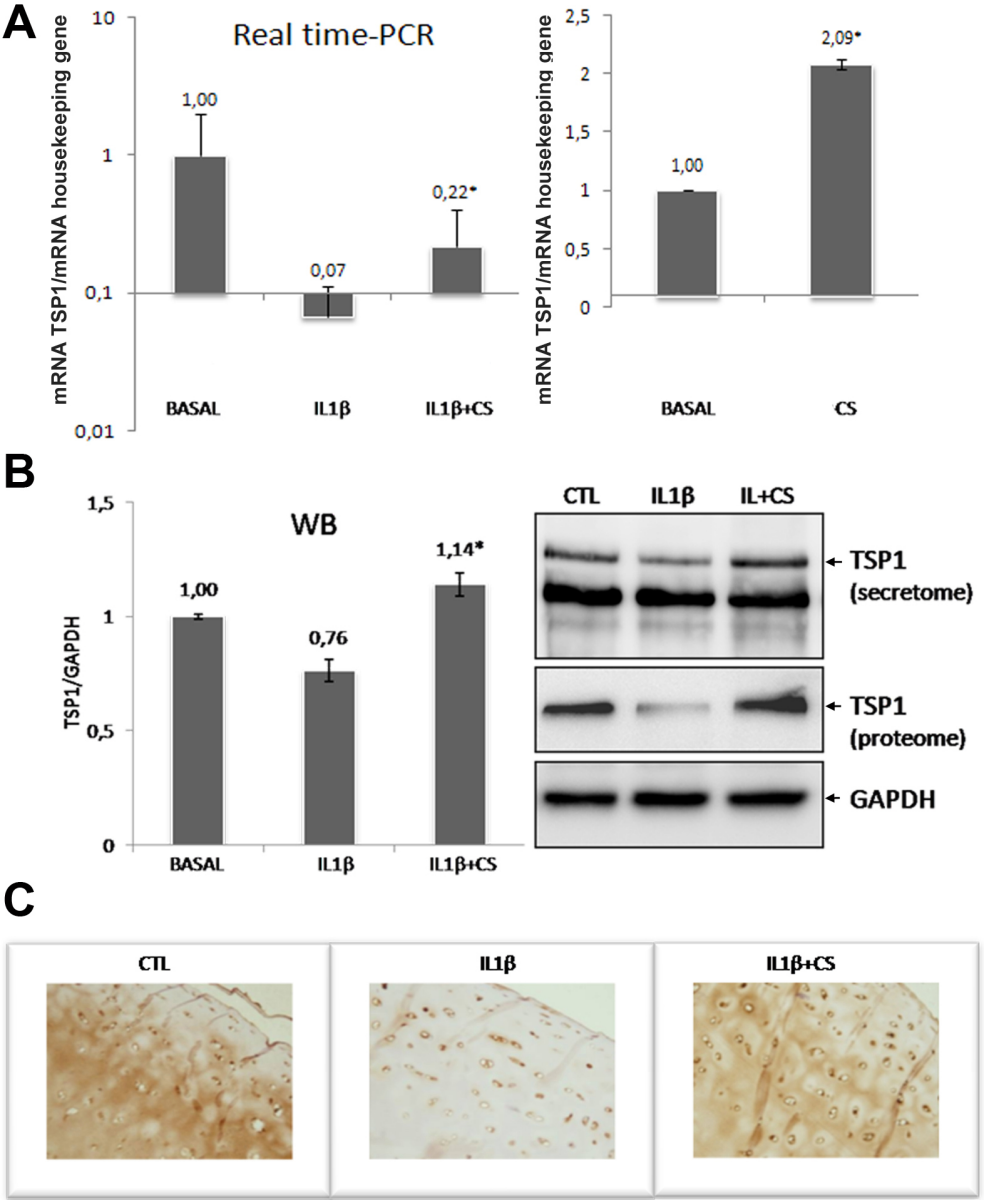
**Thrombospondin-1 (TSP1 or THBS1) is increased by chondroitin sulfate**. Overexpression values of thrombospondin-1(TSP1) were determined by **(A) **real-time PCR and **(B) **western blot (WB) analysis of both IL-1β-stimulated and unstimulated human articular chondrocytes, and by **(C) **immunohistochemical analysis of cartilage shavings as described in Materials and methods. Each condition was tested in duplicate. Results are expressed as the mean ± standard error of the mean of three independent experiments. Representative images of western blot and immunohistochemical assays are shown. *P < 0.05, CS-treated group was significantly different from IL-1β-stimulated group. CS, chondroitin sulfate; CTL, control.

The data obtained in the SILAC analysis need to be validated for differences in protein expression profiles before the biological roles of the modulated proteins are extensively studied. We therefore performed additional studies in order to verify the altered abundance of TSP1 in CS-treated chondrocytes. Interestingly, TSP1 is a multifunctional adhesive glycoprotein present in articular cartilage and synthesized by articular chondrocytes [34], whose gene transfer suppresses the disease progression of experimental OA [31]. The inhibitory effect of TSP-1 on angiogenesis has been largely described [35]. Owing to the pivotal role of angiogenesis in OA physiopathology [36], we decided to verify TSP1 gene expression level in CS-treated chondrocytes stimulated with IL-1β by real time-PCR analysis, and also in cells without cytokine stimulation. As shown in Figure 5A, CS upregulates TSP1 already in the absence of IL-1β. When the cytokine is present, CS is capable of counteracting its suppressive effect on TSP1 in chondrocytes. Furthermore, TSP1 protein levels were also evaluated in chondrocyte conditioned media (secretome) and cellular extracts (proteome) by western blot analyses and in cartilage explant culture by immunohistochemistry (Figure 5). The increase of TSP1 protein observed both in cell and tissue cultures following CS treatment suggests the possible mechanism through which this drug could exert an anti-angiogenic action.

## Conclusion

Our work provides a comprehensive quantitative analysis of the effects of CS in IL-1β-stimulated chondrocyte secretome, as well as novel molecular evidence for its anti-angiogenic, anti-inflammatory, and anti-catabolic properties. Proteins modulated by this drug are potential new targets for OA treatment (for example, TSP1). These findings might provide a rationale for targeting angiogenesis as a disease-modifying therapy for OA.

## Abbreviations

CS: chondroitin sulfate; C_t_: threshold cycle; DMEM: Dulbecco's modified Eagle's medium; ECM: extracellular matrix; GS: glucosamine sulfate; HAC: human articular chondrocyte; IL: interleukin; LC: liquid chromatography; mAb: monoclonal antibody; MALDI: matrix-assisted laser desorption/ionization; MS: mass spectrometry; OA: osteoarthritis; PCR: polymerase chain reaction; SILAC: stable isotope labeling with amino acids in cell culture; TOF: time of flight; TFA: Trifluoroacetic acid; TNF: tumor necrosis factor; TSG6: TNFα-induced protein; TSP1: thrombospondin-1.

## Competing interests

The authors declare that they have no competing interests.

## Authors' contributions

VC carried out the experimental work, analyzed the data and drafted the manuscript. LL and BR helped to collect and process protein samples, participated in western blot experiments and helped with statistical data analysis. PF-P and JM carried out the MS analysis and database search. EM and JV provided CS and helped with the study design. CR-R participated in the study design, interpretation of the data and manuscript preparation. FJB conceived and coordinated the project and revised the manuscript. All authors read and approved the final manuscript.
